# Synaptic scaling generically stabilizes circuit connectivity

**DOI:** 10.1186/1471-2202-12-S1-P372

**Published:** 2011-07-18

**Authors:** Christian Tetzlaff, Christoph Kolodziejski, Marc Timme, Florentin Wörgötter

**Affiliations:** 1 Network Dynamics Group, Max Planck Institute for Dynamics and Self-Organization, 37073, Göttingen, Niedersachsen, Germany; 2 Institute for Physics III – Biophysics, Georg-August University, 37077, Göttingen, Niedersachsen, Germany; 3 Institute for Physics, Nonlinear Dynamics, Georg-August University, 37077, Göttingen, Niedersachsen, Germany; 4 Bernstein Center for Computational Neuroscience, 37073, Göttingen, Niedesachsen, Germany

## 

Neural systems regulate synaptic plasticity avoiding overly strong growth or shrinkage of the connections, thereby keeping the circuit architecture operational. Accordingly, several experimental studies have shown that synaptic weights increase only in direct relation to their current value, resulting in reduced growth for stronger synapses [1]. It is, however, difficult to extract from these studies unequivocal evidence about the underlying biophysical mechanisms that control weight growth.

The theoretical neurosciences have addressed this problem by exploring mechanisms for synaptic weight change that contain limiting factors to regulate growth [2]. The effectiveness of these mechanisms is difficult to justify from a biophysical perspective, in particular those that require knowledge of global network status (e.g. knowledge of the ‘sum of all weights’) for normalization. Also spike-timing-dependent plasticity [3] cannot guaranty stability because various types of plasticity exist across different neurons and even at the same neuron, depending on the location of the synapses [1].

Therefore, it remains an open question how neural circuits simultaneously stabilize their many synapses and ensure diversity in the presence of a variety of distinct plasticity mechanisms.

In 1998, a series of studies initiated by Turrigiano augmented this discussion by demonstrating that network activity is homeostatically regulated, suggesting that weights *ω* are regulated by an activity-dependent difference term [4,5]. Accordingly, synaptic scaling compares output activity *v* against a desired target activity *v_T_* of each individual neuron [5]. Most straightforwardly, such a local weight change is defined by *dω*/*dt* = *γH*(*ν_T_* – *ν*) [6], where the long characteristic time scale (hours up to days) of synaptic scaling is determined by a small factor *γ* << 1. Synaptic scaling operates in parallel to conventional plasticity and acts simultaneously on different synapses. Here we suggest that synaptic scaling is combined with different types of plasticity mechanisms in the same circuit or even at the same neuron and regulates synaptic diversity across the circuit.

We demonstrate that it robustly yields stable and diverse weight distributions which moreover are independent of the individual plasticity mechanism. As scaling co-acts with plasticity, such a combined mechanism is mathematically characterized by a weight change *dω*/*dt* = *μG* + *γH*. Here *μ* defines the rate of change of conventional synaptic plasticity, *γ* <<*μ* << 1, and *G* and *H* describe the specific types of plasticity and scaling, respectively [7]. For example, *G* is different for plain Hebbian plasticity than for STDP. As we show, combining any type of conventional plasticity *G* with nonlinear weight-dependent scaling *H* naturally yields global synaptic stabilization across the circuit regardless of the specific form of the plasticity *G* and also largely independent of the intrinsic neuron dynamics. Our study demonstrates that synapses are stabilized strictly in an input-determined way thereby capturing characteristic features of the inputs to the network. As an important result, we show that such systems are capable of representing a given input pattern via stably changed weights along several stages of signal propagation. This holds even in circuits containing a substantial number of random recurrent connections but no particular additional architecture.
